# Asymptomatic traumatic ruptured intracranial dermoid cyst: a case report

**DOI:** 10.1016/j.radcr.2025.08.035

**Published:** 2025-09-11

**Authors:** Prajwol Paneru, Amrit Bhusal, Bishesh Lamichhane, Sabin Acharya, Prashanta Paneru, Alina Chapagain, Rahul Shrestha

**Affiliations:** a Department of Medicine, Gandaki Medical College Teaching Hospital and Research Center Pvt Ltd, Nayabazaar, Pokhara, 33700, Nepal; b Department of Medicine, BP Koirala Institute of Health Sciences (BPKIHS), Ghopa, Sundari, Dharan 56700, Nepal; c Department of Radiology, Nepalese Army Institute of Health Sciences College of Medicine, Bhandarkhal, Kathmandu 44600, Nepal; d Department of Medicine, Chitwan Medical College, Chaubiskothi, Chitwan, 44200, Nepal; e Department of Medicine, Tribhuvan University Teaching Hospital, Maharajgunj Medical Campus, Maharajgunj, Kathmandu 44600, Nepal; f Department of Radiology, Nepal Medical College, Attarkhel, Kathmandu 44611, Nepal

**Keywords:** Intracranial, Traumatic, Ruptured dermoid cyst, Conservative management, Case report

## Abstract

Intracranial dermoid cysts are rare congenital cystic lesions accounting for less than 0.5% of all primary intracranial tumors. They are often asymptomatic but can present with severe complications upon rupture, either spontaneously or traumatically. This report describes a unique case of a 38-year-old female with a traumatic rupture of an intracranial dermoid cyst following mild head injury. The patient was asymptomatic, with no neurological deficits on presentation or follow-up. Imaging revealed a welldefined, fat-attenuating lesion in the right cerebral hemisphere and fat droplets disseminated into the ventricular system and adjacent brain parenchyma. The patient was managed conservatively with close clinical and radiologic monitoring, avoiding surgical intervention due to the absence of symptoms. This case highlights the importance of recognizing traumatic rupture of intracranial dermoid cysts as a potential consequence of head trauma and underscores the need for individualized management to balance surgical risks against potential long-term complications.

## Introduction

Intracranial dermoid cysts are rare**,** benign congenital cystic lesions arising from epithelial cells retained during neural tube closure and comprised of elements of retained hair, sweat and sebaceous glands. These account for less than 0.5% of all primary intracranial tumors [1]. These lesions are commonly located in midline and frequently involve the posterior fossa and suprasellar region [2]. While typically slow-growing and asymptomatic, dermoid cysts can become clinically significant when they rupture. However, rupture of intracranial dermoid cyst is rare**.** Rupture may occur spontaneously due to gradual accumulation of internal contents or, more rarely, as a result of trauma [3]. Rupture presents with headache (57%) as the most common symptom followed by seizures (42%) and obstructive hydrocephalus (29%) [4]. Here, we report a rare case of a 38-year-old female who was incidentally diagnosed with a ruptured intracranial dermoid cyst following minor head trauma. Uniquely, the patient remained entirely asymptomatic throughout the course of evaluation and follow-up. The case highlights the diagnostic and management considerations for this uncommon presentation and highlights the importance of careful imaging review, even in neurologically intact trauma patients.

## Case presentation

A 38-year-old female presented to the Emergency Department (ED) following a low-velocity road traffic accident as a pillion rider with mild head injury. She reported hitting her head on the road but did not lose consciousness**.** The patient denied complaints of headache, vomiting, loss of consciousness, seizures and limb weakness at any point before or after the trauma. Her medical history was unremarkable. She had no known history of chronic illnesses such as hypertension, diabetes mellitus, or epilepsy. Surgical history was negative, and she was not on any regular medications. There was no history of prior head trauma, brain imaging, or neurological symptoms. Family history was negative for neurological or congenital disorders. She was a nonsmoker, did not consume alcohol, and denied illicit drug use. On presentation, the patient’s vital signs were stable with blood pressure: 132/86 mmHg, heart rate: 82 bpm, respiratory rate: 16/min, temperature: 36.8°C, SpO₂: 99% on room air. Glasgow Coma Scale was 15/15. A thorough neurological examination revealed normal findings with no focal deficits. Cranial nerves were intact. Motor and sensory examinations were within normal limits. Cerebellar signs and gait were unremarkable. Laboratory investigations were within normal limits. Complete blood count (CBC) showed:

➢ Hemoglobin: 12.9 g/dL (normal: 12-16 g/dL)
➢ White blood cell count: 6,500 /µL (normal: 4,000-11,000 /µL)
➢ Platelets: 252,000 /µL (normal: 150,000-400,000 /µL)

Electrolytes, renal function tests, liver function tests, and coagulation profile were all within normal reference ranges. Blood glucose was 88 mg/dL (normal: 70-110 mg/dL). No signs of systemic infection or metabolic imbalance were detected.

**Fig. 1 (A):**
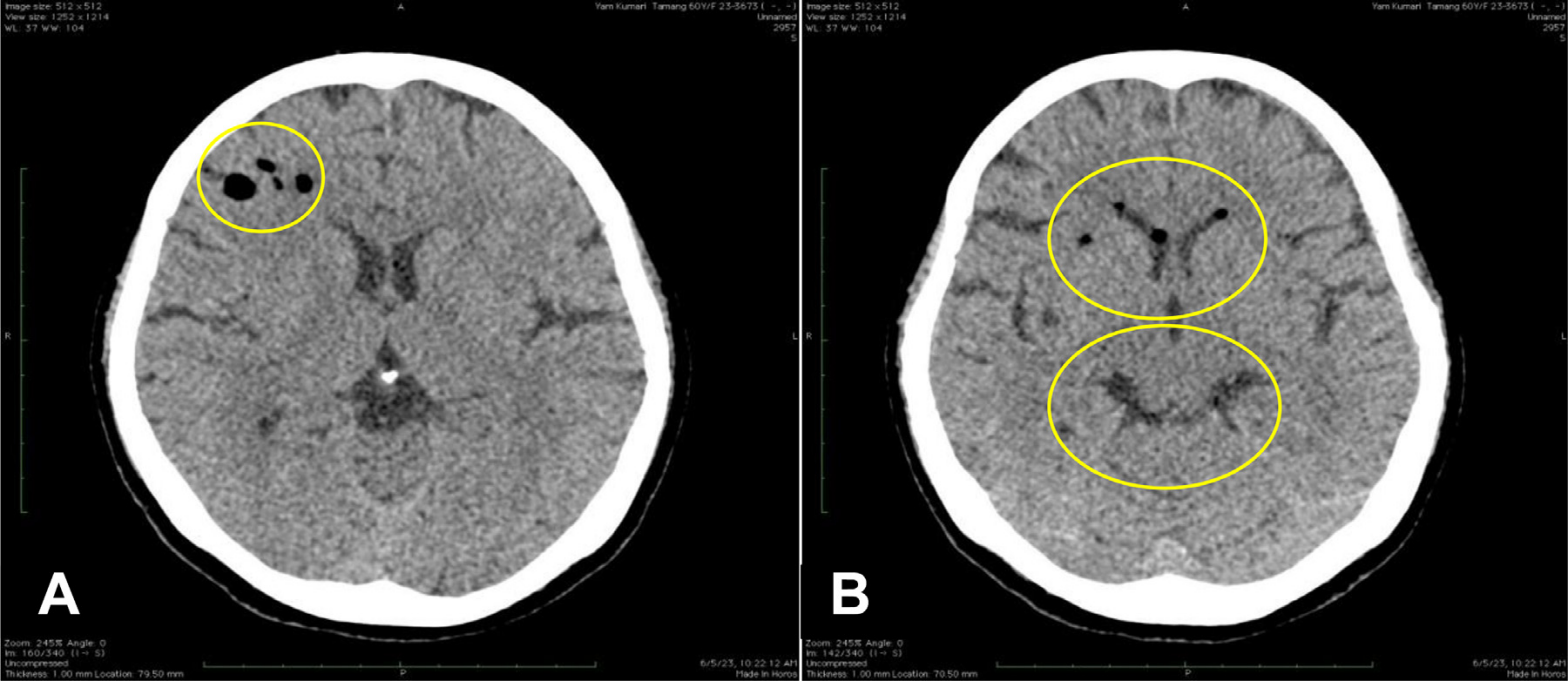
NCCT head showing well defined, round to oval fat attenuating lesion in right cerebral hemisphere (marked by circle). (B) NCCT head showing fat attenuating lesions in frontal horns of bilateral lateral ventricles and basal cistern (marked by double circles).

A noncontrast computed tomography (NCCT) of the head, performed on the same day of presentation, revealed a well-defined, round to oval fat-attenuating lesion in the right cerebral hemisphere, specifically in the basisphenoid region (marked by circle in Fig. 1A). Additionally, multiple small fat-attenuating lesions were seen disseminated within the frontal horns of bilateral lateral ventricles and basal cistern, indicative of rupture (marked by circle in Fig. 1B). There was no associated hemorrhage, hydrocephalus, midline shift, or mass effect observed.

Given the imaging findings and absence of symptoms, the patient was managed conservatively without any surgical and medical treatment. She was observed in the ED for 24 hours, during which she remained hemodynamically stable and neurologically intact. She was discharged with advice for early follow-up and return if symptoms developed.

**Fig. 2 (A):**
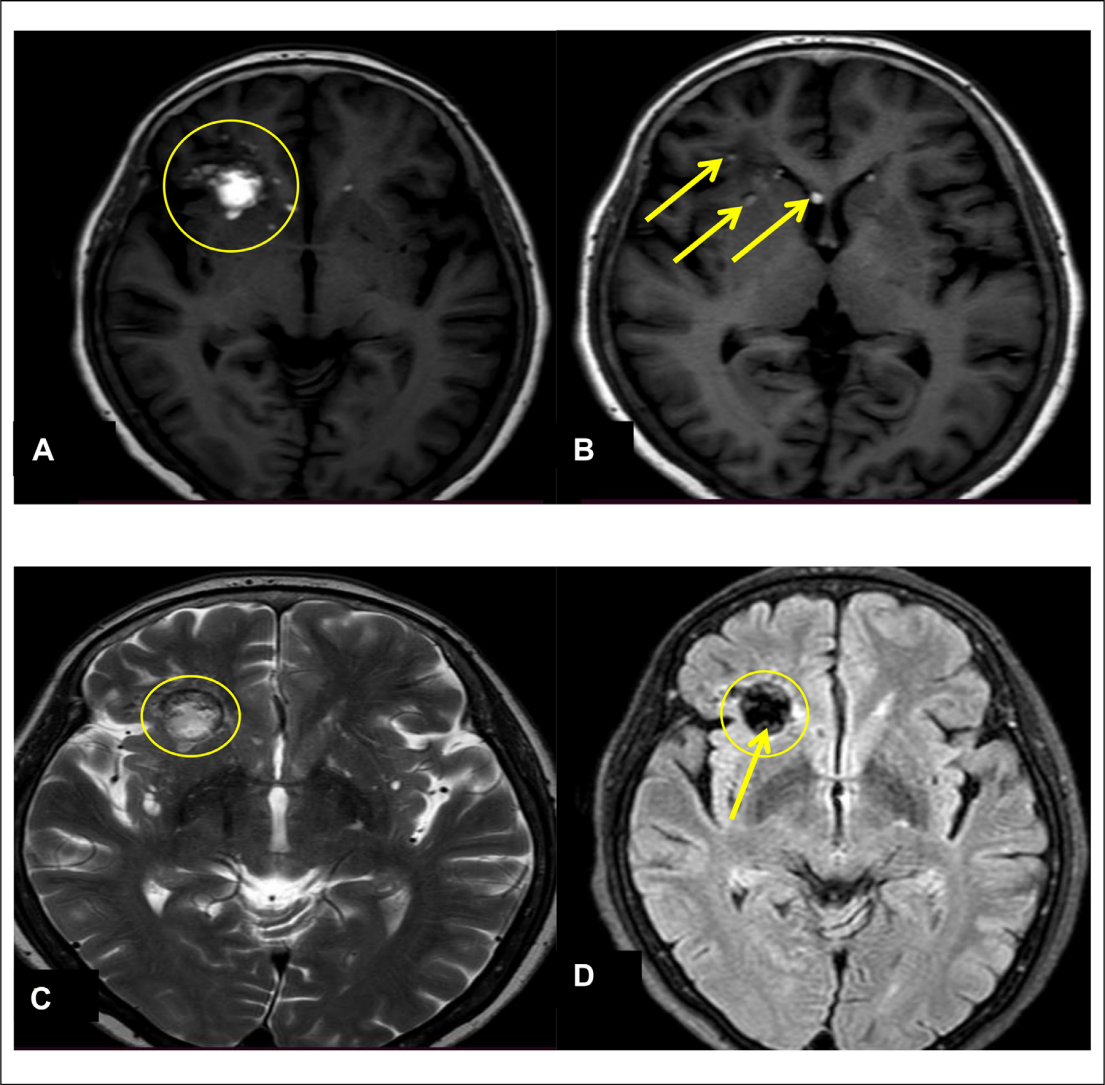
MRI brain (T1 weighted axial MR sequence) showing a well-defined, round to ovoid lesion measuring approximately 19×18×24mm (APxTRxCC) in right basisphenoid appearing intensely hyperintense (marked by circle). (B) Few disseminated hyperintense droplets in adjacent brain parenchyma, sulcal spaces and in frontal horns of bilateral lateral ventricles (marked by arrows). (C) Heterogeneously hyperintense lesion in right basisphenoid region was revealed in T2-weighted sequence (marked by circle). (D) Heterogeneously hypointense lesion noted in FLAIR image representing fatty components (marked by arrow).

A follow-up Magnetic Resonance Imaging (MRI) of the brain was performed 2 weeks later. T1-weighted axial MR sequences revealed a hyperintense well-defined, round to ovoid lesion measuring approximately 19 × 18 × 24mm (anteroposterior × transverse × craniocaudal) in right basisphenoid, consistent with a dermoid cyst, due to lipid content (marked by circle in Fig. 2A). Additionally, multiple small hyperintense foci were visualized in adjacent brain parenchyma, sulcal spaces and in frontal horns of bilateral lateral ventricles (marked by arrow in Fig. 2B). Heterogeneously hyperintense lesion in right basisphenoid region was revealed in T2-weighted sequence (marked by circle in Fig. 2C). Heterogeneously hypointense lesion was noted in FLAIR image representing fatty components (marked by arrow in Fig. 2D) which is classical for dermoid cysts with high fat content. No signs of perilesional edema, enhancement, hydrocephalus, or mass effect were noted.

With above imaging findings, diagnosis of asymptomatic traumatic rupture of intracranial dermoid cyst was made. No medications were prescribed as the patient remained asymptomatic. She was counseled in detail about the diagnosis, potential complications including chemical meningitis**,** hydrocephalus, or seizures, and the patient was advised to consult with neurosurgeon. Surgical intervention was deferred given the absence of symptoms, widespread lipid droplet distribution, and high risk of morbidity and was followed monthly for 6 months. The danger signs and possible complications were explained to the patient and her family members. During the follow up visits, the patient reported no symptoms pertaining to the diagnosis and was again advised to do an MRI brain at 3 and 6 months, but the patient was reluctant to do so due to financial issues.

## Discussion

Intracranial dermoid cysts are rare**,** benign congenital tumors arising from ectodermal inclusions during neural tube closure typically between the 5th to 6th weeks of fetal life [5]**.** These lesions often remain asymptomatic unless they rupture, either spontaneously or following head trauma. Ruptured ICD cysts usually present with symptoms related to mass effect like headaches, seizures, or focal neurological deficits, or due to chemical meningitis following spontaneous rupture [6]. Traumatic rupture of intracranial dermoid cysts is exceedingly rare. Only a limited number of cases have been reported in the literature [7]. Spontaneous rupture generally occurs due to gradual accumulation of internal cystic contents, leading to increased intracystic pressure. In contrast, trauma-induced rupture likely results due to shearing and damage of cyst wall with symptoms depending upon the site of lesion and the dissemination of lipid droplets [8].

The most striking feature in this case is the absence of any neurological symptoms despite imaging evidence of rupture and dissemination of fat droplets. Despite the initial NCCT Head revealing a ruptured dermoid cyst with fat attenuating lipid droplets scattered over the adjacent brain parenchyma and ventricular system, the patient was asymptomatic and intact neurologically. There could be several possible explanations for this lack of symptoms in our patient. Firstly, the location of the cyst and pattern of lipid dissemination could have avoided critical neurovascular structures or areas involved in overt neurological function. Secondly, the disseminated fat droplets may not have triggered an inflammatory or chemical meningitis response. Furthermore, the variability among individuals in the brain's response to a foreign body could also be at play. This case point to the potential of fairly minor head trauma to result in a significant intracranial consequence, particularly where there is pre-existing though unrecognized lesion.

Incidental diagnosis on imaging for an unrelated trauma of a ruptured dermoid cyst carries important consequences to the management of such a case. The patient was initially asymptomatic, but with the discovery of breakdown products of lipids in the cerebrospinal fluid, the risk for late complications, such as chemical meningitis, obstructive hydrocephalus, or even vasospasm exists [4]. Ramlakhan R. [1] reported a traumatic rupture presenting with seizures, necessitating surgical intervention**.** Aktham et al*.* [2] reported a similar case where traumatic rupture of a dermoid cyst led to continued lipid excretion and migration into the ventricles and subarachnoid space over time followed by sustained deposition on prolonged radiological follow up of patient. Likewise, Zhang et al. [8] reported a case of asymptomatic traumatic rupture, which was also managed conservatively with favorable short-term outcomes**.** Surgical resection is the definitive treatment for symptomatic dermoid cysts to prevent recurrence and alleviate mass effect [6]. The optimal long-term management of incidentally discovered asymptomatic ruptured dermoid cysts remains controversial. With asymptomatic presentation, however, risks of surgery, like resulting neurological impairment, must be weighed against possible benefits of complete resection of the cyst [1]. In this particular case, the patient's initial asymptomatic presentation and scattered nature of lipid droplets after rupture may make total surgical excision technically challenging and perhaps with increased morbidity. Therefore, conservative management with close follow-up was done and patient was counseled regarding potential delayed symptoms.

However, there are important limitations to this case report. Follow-up imaging beyond 2 weeks was not performed due to financial constraints, and thus, the long-term natural history of the lesion remains uncertain. Additionally, the possibility of delayed onset symptoms or complications cannot be ruled out, emphasizing the need for ongoing surveillance.

This case contributes to the limited literature regarding asymptomatic traumatic rupture of intracranial dermoid cysts and highlights the importance of retaining this rare entity as a consideration for post-traumatic imaging findings, even in the absence of immediate neurological deficits. It also highlights the importance of close clinical and radiologic follow-up in such cases to detect and manage potential delayed complications. Further studies and case accumulation are necessary to fully appreciate the natural history and optimal management of asymptomatic traumatic ruptured intracranial dermoid cysts.

## Conclusion

Traumatic ruptured intracranial dermoid cyst is a rare occurrence and only a handful of cases have been reported in literature till date. Ruptured ICD cyst usually presents with headache and/or seizures; however, we report a case of an asymptomatic ruptured ICD cyst that was managed conservatively and followed 2 weeks later with no significant deterioration on clinical and radiological evaluation. Although complete surgical resection of cyst is optimal, it should be deferred until absolutely necessary to minimize the damage to adjacent vital structures and followed over time to evaluate progression of lesion and neurological status.

## Ethical approval

The study is exempt from ethical approval in our institution.

## Provenance and peer review

Not commissioned, externally peer-reviewed.

## Role of generative AI

None.

## Patient consent

Written informed consent was obtained from the patient for publication of this case report and accompanying images. A copy of the written consent is available for review by the Editor-in-Chief of this journal on request.
